# The mediating role of risk perception in the association between industry-related air pollution and health

**DOI:** 10.1371/journal.pone.0196783

**Published:** 2018-05-03

**Authors:** Arnold D. Bergstra, Bert Brunekreef, Alex Burdorf

**Affiliations:** 1 Department of Public Health, Erasmus MC, University Medical Centre, Rotterdam, the Netherlands; 2 The Zeeland Public Health Service, Goes, the Netherlands; 3 Institute for Risk Assessment Sciences, Utrecht University, Utrecht, the Netherlands; 4 Julius Center for Health Sciences and Primary Care, University Medical Center Utrecht, Utrecht, the Netherlands; Universite de Bretagne Occidentale, FRANCE

## Abstract

**Background:**

Heavy industry emits many potentially hazardous pollutants into the air which can affect health. Awareness about the potential health impacts of air pollution from industry can influence people’s risk perception. This in turn can affect (self-reported) symptoms. Our aims were to investigate the associations of air pollution from heavy industry with health symptoms and to evaluate whether these associations are mediated by people’s risk perception about local industry.

**Methods:**

A cross-sectional questionnaire study was conducted among children (2–18 years) and adults (19 years and above) living in the direct vicinity of an area with heavy industry. A dispersion model was used to characterize individual-level exposures to air pollution emitted from the industry in the area. Associations between PM_2.5_ and NO_X_ with presence of chronic diseases (adults) and respiratory symptoms (adults and children) were investigated by logistic regression analysis. Risk perception was indirectly measured by worries about local industry (0–10 scale). Mediation analyses were performed to investigate the role of mediation by these worries.

**Results:**

The response was 54% (2,627/4,877). In adults exposure to modelled PM_2.5_ from industry (per μg/m^3^) was related with reported high blood pressure (OR 1.56, 95% CI 1.13–2.15) and exposure to modelled NO_X_ (per μg/m^3^) was inversely related with cardiovascular diseases (OR 0.91, 95% CI 0.84–0.98). In children higher PM_2.5_ and NO_X_ concentrations (per μg/m^3^) were related with wheezing (OR 2.00, 95% CI 1.24–3.24 and OR 1.13, 95% CI 1.06–1.21 respectively) and dry cough (OR 2.33, 95% CI 1.55–3.52 and OR 1.16, 95% CI 1.10–1.22 respectively). Parental worry about local industry was an important mediator in exposure–health relations in children (indirect effect between 19–28%).

**Conclusion:**

Exposure from industry was associated with self-reported reported high blood pressure among adults and respiratory symptoms among their children. Risk perception was found to mediate these associations for children.

## Introduction

Air pollution is a complex mixture of different gaseous and particulate components and can cause several health effects. Both long- and short-term exposure to air pollution can cause cardiovascular, respiratory diseases (e.g., asthma, chronic obstructive pulmonary disease, lung cancer) and mortality [1]. In addition to the diseases listed above air pollution (PM_2.5_) can also cause some physiological changes as blood pressure [2].

Children and adolescents are more susceptible to the effects of air pollution than adults. The lack of a fully developed pulmonary metabolic capacity in children make them more susceptible to air pollutants compared with adults [3]. Moreover children are in general more exposed because of the greater activity of children compared with adults, as well a greater time spent outdoors.

Investigations often focus on emissions from road traffic, smog and urban or regional differences in air pollution. Research about air pollution from heavy industry on health is less often explored [4–6]. The impact of localised air pollution from industry on health is a major concern in some areas.

Some studies have found an increased risk of hospitalization for cardiorespiratory complaints in adults [4,7] and children [5–7] living close to industrial plants while others show no such relationships in adults [8]. Questionnaire studies on respiratory effects in children living in the vicinity of industrial plants are rare. A cross-sectional study of children (6–12 years) living close to petrochemical plants in La Plata, Argentina had significant more asthma (25% vs 10%), asthma exacerbations (6.7 vs 2.9 per year) and respiratory symptoms, such as current wheeze, dyspnea, nocturnal cough or rhinitis (average 24% vs 14%) than those living in the semirural control regions [7]. In a cross-sectional study among children aged 11 to 14 years attending schools near a petrochemical refinery in Cape Town, South Africa respiratory symptom were more prevalent than in children in other areas of the city, with frequent waking with wheezing being in great excess (OR 8.92, 95% CI 4.79–16.63) [9].

In a cross-sectional study of wheezing in children and adolescents (aged 0 to 14 years) living in the vicinity of the Guamaré petrochemical complex in Brazil found statistically significant associations between wheezing in the past 12 months and living in exposed communities versus reference communities (OR 2.01, 95% CI 1.01 to 4.01) [10].

Risk perception towards industry can possibly influence the association between industrial pollution and health symptoms. People with a high risk perception are likely to report more health symptoms. Also, a high risk perception may act like a stressor and therefore can influence people’s health. Chemical odours may strengthen the risk perception due to people’s concerns about their health. In other words, exposure—health outcomes association can be mediated by odour and worry. In the presence of a strong mediating effect of risk perception reported associations between industrial air pollution and health problems among citizens in the vicinity of industry may be spurious. Some epidemiological studies have indeed presented indications that (risk and odour) perception mechanisms partly explain the increased occurrence of self-reported health symptoms among residents living close to chemical industry [11–14]. However, these studies have not formally addressed the magnitude or direction of the mediating role of risk perception.

To the best of our knowledge, studies about the mediating role of risk perception in the association between industry-related air pollution and health among adults and their children are very rare. Therefore, the aims of this study were to estimate (1) the association between air pollution from industry and health symptoms and (2) the mediating role of risk perception about local industry in the association with health symptoms.

## Materials and methods

### Study design and population

A cross-sectional study was conducted among inhabitants of the seven villages (postal code areas) in the direct vicinity (2–5 km) of the large industrial area (Sloe area) near East Vlissingen in the Southwest of the Netherlands. A representative total sample of 4877 inhabitants, aged 19 years and above, were randomly recruited in each of the seven villages (an average of 697 potential respondents per village). At the time of the study several heavy industries were active in the area such as a coal power plant, terminals for storing and shipping of coal, a plastic recycling company, a phosphorus chemical company, an oil refinery and an aluminium smelter.

The selected inhabitants received an invitation letter and paper questionnaire by mail. The participants could return the questionnaire in a postage-paid envelope that was included in the mail package or they could complete the questionnaire online by using a log in code provided in the letter. Two reminders were sent two and four weeks later in case of non-response. In the first reminder the log in code was included. In the second reminder, in addition to the login code, the paper questionnaire was also included. Both the paper questionnaire and the internet version of the questionnaire were distributed and collected by an external company. The data were collected from 20 June 2011 to 22 August 2011.

In this study, with secondary data analysis, we have used data collected by The Zeeland Public Health Service. All municipal Public Health Services in the Netherlands are required by law to gain insight into the health of the local population. The written information to participants explained that by filling out the questionnaire informed consent was given for use of data for research purposes. Under the Dutch law for medical scientific research with human subjects questionnaire surveys are not subject to approval by an institutional ethics committee. The Law for Protection of Personal Data requires informed consent and also procedures for the protection of personal privacy. These procedures are laid down in the Code of Conduct for Medical research (at www.federa.org), established by the Council of the Federation of Medical Scientific Societies. These procedures were strictly adhered and the data were analysed anonymously.

### Exposure assessment

A variety of components are emitted by plants in the industrial area near East Vlissingen in the Southwest of the Netherlands like particulate matter (PM), nitrogen oxide (NO_X_), sulphur dioxide (SO_2_), ethene, formaldehyde, toluene, benzene, and dioxins. It is difficult to define an exposure measure of relevance when the biological mechanisms are largely unknown. Moreover the air pollution mix varied greatly by locality and time [15]. For this study the compounds were selected by following two steps. First, the emission (kg/year) of a compound was divided by the European Commission limit values or if not available the maximum permissible risk levels (MPR) in air (μg/m^3^) from the National Institute for Public Health and the Environment, The Netherlands (RIVM). The compounds with a high fraction (more than 5.000) were selected. Next, the annual mean concentration of these compounds were calculated with a dispersion model. The compounds with a high concentration variation (mean/SD ≈ 2) were selected.

The emission data was obtained from the Emission Register [16]. The National Institute for Public Health and the Environment (RIVM) co-ordinates the annual compilation of the Emission Register on behalf of the Ministry of Infrastructure and Environment. Emission factors are derived from measurements and calculations of a model or (the international) literature.

The Operational Priority Substances (OPS) dispersion model (version 4.5.0), developed by the Netherlands National Institute for Public Health and the Environment (RIVM), was used to calculate concentration levels at individual homes. The OPS model requires emission data (emission strength, emission height, coordinates source, heat capacity and substance) and hourly-based meteorological data (among others: temperature, relative humidity, wind speed, wind direction, precipitation and global solar radiation) as input for the calculations. The meteorological data were retrieved from the Royal Netherlands Meteorological Institute (KNMI). The OPS model also requires a receptor file. The geographic information system QGIS (version 2.18.0) was used to geocode (by means of a plugin) the home addresses of the respondents. The x,y coordinates of the home addresses were used for the receptor file in the OPS model. After the dispersion calculations the air pollution data were linked to the questionnaires by means of a Trusted Third Party.

After applying the two selection steps the following compounds remained: PM_2.5_ (particulate matter with an aerodynamic diameter < 2.5 μm), PM_10_ (particulate matter with an aerodynamic diameter < 10 μm), SO_2_, and NO_X_. These compounds were highly correlated (Pearson correlation coefficients ranged from 0.67 to 0.998). Because of the high correlation, associations between questionnaire reports and these four components cannot be disentangled. Therefore PM_2.5_ was chosen because it varied most of the four compounds and NO_X_ because of the lowest correlation with PM_2.5_. Because of the high correlations with other components PM_2.5_ and NO_X_ must be regarded as indicators of the mixture of air pollution rather than the causative factors of adverse health effects.

In the study area the exposure to air pollution form traffic is relatively low (less than 5,000 vehicles per day or the distance between road and house is more than 100 meters). Only three (trunk) roads (A58, N62 and N254) have more than 5,000 vehicles per day (35,000. 18.000 and 12,000 vehicles per day respectively) and the distance between these roads and responders home address was less than 100 meters for only approximately 30 households. Therefore, traffic-related air pollution will not be an important potential confounder and, thus, was not included in the study.

### Questionnaire for adults on health and perception

The questionnaire for adults consisted of four parts namely: socio-demographic characteristics, health problems, health behaviour, and perception.

#### Socio-demographic

Socio-demographic characteristics were gender, age (19–34, 35–49, 50–64 and more than 65 years) and education. The question about education was categorized in: 1) primary school or less (8 years of education or less), 2) lower general secondary education (12 years of education), 3) higher general secondary education (14 years of education) and 4) college or university (more than 14 years of education).

#### Health problems

The health problems related to general health and chronic disease(s). General health was measured with the question: How would you describe your health? Five answers were possible: 1) excellent, 2) very good, 3) good, 4) fair and 5) poor [17]. Ill health was defined by fair or poor health, with as reference category good to excellent health.

Respondents were asked about the presence of chronic disease(s) in the past 12 months. Among others, one of more of the following categories could be selected: a) cerebral infarction, Transient Ischemic Attack (TIA) or stroke, b) heart attack, c) another serious heart disease (e.g. heart failure or angina pectoris), d) high blood pressure and e) respiratory diseases (asthma, chronic bronchitis, pulmonary emphysema or chronic obstructive respiratory diseases). Respondents could also fill in whether the disease has been diagnosed by a doctor. The selection of chronic diseases was based on a comprehensive list of chronic diseases by Statistics Netherlands for use in community-based studies [18].

#### Health behaviour

Smoking was assessed by asking how many cigarettes on average per day were smoked. Passive smoking was assessed by whether another family member smoked in house.

#### Perception

Risk perception was indirectly measured by asking the respondents to rate their level of concern about potential impacts of the industry in their neighbourhood on their health and well-being in the past 12 months. A 11 point scale (0: not worried at all, 10: extreme worried) in a single question was used. In all statistical analyses this variable was used as a continuous variable. It has been suggested that a respondent can be classified as very worried if he or she rates a value of seven or higher on the risk scale [19].

Respondents were asked to rate their level of odour annoyance about industrial odour when they are in or around their home in the past 12 months. A 11 point scale (0: no annoyance at all, 10: extreme annoyance) in a single question was used. In all statistical analyses this variable was used as a continuous variable. A respondent was classified as very annoyed if he or she rates a value of seven or higher on the annoyance scale.

### Questionnaire about the health of the children

Parents were asked to fill out questions about the health of their children (2–18 years) with particular focus on respiratory symptoms. The questionnaire about the children consisted of three parts namely: demographic characteristics, health problems and health behaviour.

#### Demographic

Demographic characteristics were gender and age (2–5, 6–9, 10–13 and 14–18 years). The groups are composed in way that there are about the same number of children in each group

#### Health problems

For the questionnaire about the health of the children the core questions from the International Study on Asthma and Allergies in Children (ISAAC) was used [20–22] with minor changes. These questions were 1) Has your child had wheezing or whistling in the chest in the past 12 months? 2) In the past 12 months, has your child's chest sounded wheezy during or after exercise? 3) In the past 12 months, has your child had a dry cough at night, apart from a cough associated with a cold or a chest infection?. Reported ‘‘asthma” was defined from the question ‘‘Has your child had asthma in the past 12 months?”.

#### Health behaviour

Smoking was assessed by asking whether the child smoked every day, more than once a week (but not every day), less than once a week, or did not smoke.

### Statistical analyses

Mediation analyses, according the method of Baron and Kenny [23], were carried out to examine the role of worry as mediating variable between air pollution and health outcomes. First, associations between the independent variable (air pollution) and the dependent health outcomes were analysed by logistic regression analysis (the total effect). Second, the association between air pollution (PM_2.5_ in μg/m^3^ and NO_X_ in μg/m^3^) and worry (0–10 scale) was analysed by linear regression analysis. Finally, the associations between air pollution and the health outcomes were adjusted for worry in the logistic regression analysis (the direct effect).

Mediation analysis was performed when the following criteria were met: 1) exposure must be significantly associated with the health outcomes, 2) exposure must be significantly associated with the mediator worry, 3) mediator must be significantly associated with the health outcomes.

The coefficient of the linear and logistic regressions were made comparable across the equations by multiplying each coefficient by the standard deviation (SD) of the predictor variable in the equation and then dividing by the SD of the outcome according to equations from MacKinnon and Dwyer [24]. The proportion of the direct effect was estimated by dividing the estimated direct effect by the total effect.

A moderator variable influences the strength of a relationship between two other variables. Potential moderators (gender, age, education) and confounders (smoking and passive smoking) were integrated in all multivariate analyses.

The statistical analyses were conducted with the statistical package IBM SPSS version 21 (SPSS Inc., Chicago, IL, USA). Results are presented with 95% confidence intervals (CI). A p value less than 0.05 was considered to be statistically significant.

## Results

Questionnaires were sent to 4,877 adults (19 years and more). An overall response of 54% (2627 completed questionnaires) was achieved after two reminders. For 2,477 adult respondents, the air pollution data was successfully linked to their respective home address by means of a Trusted Third Party. Parents filled out questions about the health of their children at home for 1,099 children (2–18 years). Table 1 shows the characteristics of the adult respondents and their children.

**Table 1:** Characteristics of adult respondents (19 years and more) and their children (2–18 years).

Characteristic	Adults	Children
	(n = 2477)	(n = 1099)
**Gender, n (%)**		
Man	1100 (44)	586 (53)
Women	1377 (56)	513 (47)
**Age groups (years), n (%)**		
2–5	-	249 (23)
6–9	-	277 (25)
10–13	-	287 (26)
14–18	-	286 (26)
19–34	459 (19)	-
35–49	669 (27)	-
50–64	848 (34)	-
65 and older	501 (20)	-
**Education level, n (%)**		
Primary school	182 (8)	-
Lower general secondary education	880 (37)	-
Higher general secondary education	865 (36)	-
College, university	(461) 19	-
**Smoking Status, n (%)**		
Smoker	(501) 21	40 (4)
Passive smoking in house	202 (8)	224 (21)
**Perception, mean (SD)**		
Very worried about industry in the neighbourhood	0.28 (0.39)	-
Very odour annoyed by industrial in the neighbourhood	0.19 (0.45)	-

The response differed only slightly among the adults in the seven villages with different distance to the industries. A higher response was observed among female than male responders (55% versus 45% respectively) and older age (65 years and more) than younger age (19–34 years) (70% and 44% respectively).

When using the suggested cut-off value of 7 or higher, the prevalence of being very worried about industry in the neighbourhood (28%) was significantly higher than the prevalence of being very annoyed by odour in the neighbourhood (19%) (p<0.001). The Spearman rank correlation between worried about industry in the neighbourhood and odour annoyance by industry in the neighbourhood was 0.62.

Table 2 shows that among adults the most common reported disease was high blood pressure followed by respiratory diseases, and cardiovascular diseases. The most common reported respiratory symptom among children was dry cough followed by wheezing, wheezing during exercise, and asthma.

**Table 2:** Occurrence of self-reported general health and diseases in adults (19 years and more) and their children (2–18 years).

Health/Disease	Adults	Children
	n (%)	n (%)
Fair/poor health	357 (15)	-
Cardiovascular diseases^a^	120 (5)	-
High blood pressure	501 (21)	-
Respiratory diseases^b^	224 (9)	-
Wheezing	-	164 (15)
Wheezing during exercise	-	85 (8)
Asthma	-	83 (8)
Dry cough	-	248 (23)

^a^ Cerebral infarction, TIA, stroke or other heart diseases.

^b^ Asthma, chronic bronchitis, pulmonary emphysema or chronic obstructive respiratory diseases.

The mean annual exposures originating from PM_2.5_, PM10, NO_X_ and SO_2_ emissions by industry in the study area were 0.94 (SD = 0.42), 0.68 (SD = 0.33), 5.36 (SD = 2.74) and 3.80 (SD = 1.95) μg/m^3^ respectively (without background concentration).

**Fig 1:**
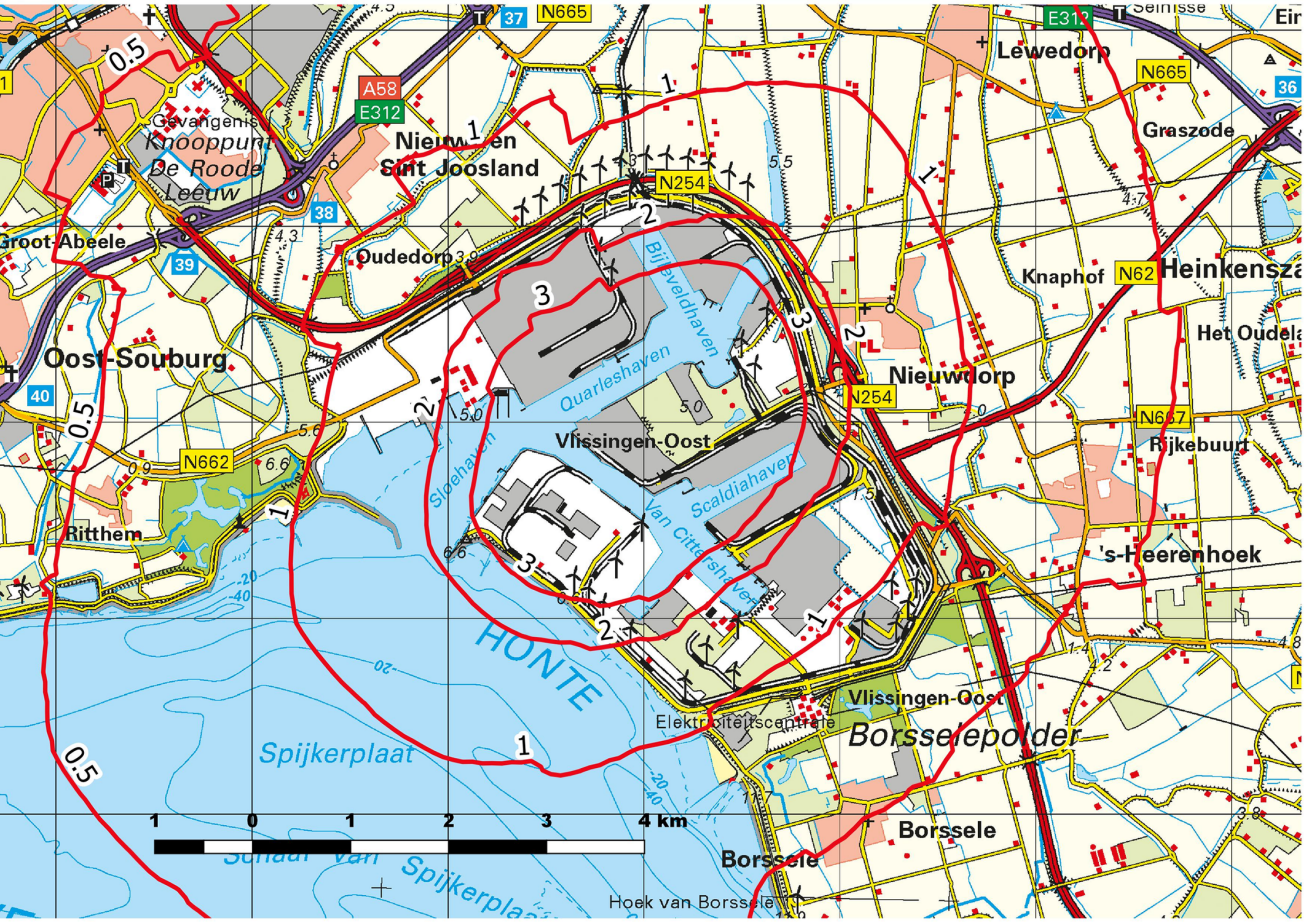
Modelled PM_2.5_ isoconcentration contours (μg/m^3^), mean annual exposure without background concentration. **Map reprinted from Kadaster in the Netherlands** [25] **under a CC-BY-4.0 license, 2017**.

Fig 1 and S1 Fig show the iso-concentration contours of the PM_2.5_ and NO_X_ contribution by industry (without background concentration) respectively.

Table 3 shows the associations between exposure and worry with measures of health by adults. Adult exposed to PM_2.5_ (per μg/m^3^) were significant more likely to report high blood pressure, after controlling for worry (OR 1.60, 95% CI 1.15–2.22). Adults exposed to NO_X_ (per μg/m^3^) were significant less likely to report cardiovascular diseases, after controlling for worry (OR 0.89, 95% CI 0.82–0.97).

**Table 3:** Associations between the exposure and worry with measures of health by adults (19 years and more) from logistic regression.

		Fair/poor health	Cardiovascular	High blood	Respiratory
			diseases^b^	pressure	diseases^c^
		OR (95% CI)^a^	OR (95% CI)^a^	OR (95% CI)^a^	OR (95% CI)^a^
PM_2.5_ (μg/m^3^)		1.04 (0.72–1.50)	0.57 (0.28–1.17)	1.56 (1.13–2.15)**	1.13 (0.74–1.73)
PM_2.5_ adjusted for worry		0.95 (0.64–1.39)	0.64 (0.30–1.34)	1.60 (1.15–2.22)**	0.99 (0.63–1.55)
Worry adjusted for PM_2.5_		1.07 (1,03–1,11)**	1.02 (0,96–1,09)	0.98 (0,95–1,02)	1.08 (1,03–1,13)**
NO_X_ (μg/m^3^)		1.01 (0.97–1.06)	0.91 (0.84–0.98)*	1.02 (0.98–1.06)	1.03 (0.98–1.08)
NO_X_ adjusted for worry		1.00 (0.95–1.05)	0.89 (0.82–0.97)*	1.02 (0.98–1.07)	1.01 (0.95–1.06)
Worry adjusted for NO_X_		1.07 (1.03–1.11)**	1.03 (0.97–1.10)	0.99 (0.95–1.02)	1.08 (1.03–1.13)**

^a^ Adjusted for gender, age, education, smoking and passive smoking.

^b^ Cerebral infarction, TIA, stroke or other serious heart diseases.

^c^ Asthma, chronic bronchitis, pulmonary emphysema or chronic obstructive respiratory diseases.

*P<0.05

** p<0.01

Table 4 shows the associations between exposure and parental worry with measures of health among children. Parents with children exposed to PM_2.5_ (per μg/m^3^) and NO_X_ (per μg/m^3^) were significant more likely to report wheezing (OR 1.71, 95% CI 1.04–2.80 and OR 1.11, 95% CI 1.03–1.18 respectively) and dry cough (OR 1.90, 95%CI 1.25–2.90 and OR 1.13, 95% CI 1.07–1.19 respectively) among their children, after controlling for worry.

**Table 4:** Associations between the exposure and worry with measures of health by children (2 to 18 years) from logistic regression.

		Wheezing	Wheezing during	Asthma	Dry cough
			exercise		
		OR (95% CI)^a^	OR (95% CI)^a^	OR (95% CI)^a^	OR (95% CI)^a^
PM_2.5_ (μg/m^3^)		2.00 (1.24–3.24)**	1.87 (1.01–3.44)*	1.15 (0.61–2.19)	2.33 (1.55–3.52)**
PM_2.5_ adjusted for worry		1.71 (1.04–2.80)*	1.56 (0.83–2.94)	1.04 (0.54–2.01)	1.90 (1.25–2.90)**
Worry adjusted for PM_2.5_		1.09 (1.04–1.16)**	1.10 (1.02–1.18)*	1.05 (0.98–1.13)	1.11 (1.06–1.17)**
NO_X_ (μg/m^3^)		1.13 (1.06–1.21)**	1.13 (1.04–1.22)**	1.00 (0.92–1.09)	1.16 (1.10–1.22)**
NO_X_ adjusted for worry		1.11 (1.03–1.18)**	1.10 (1.01–1.20)*	0.98 (0.90–1.07)	1.13 (1.07–1.19)**
Worry adjusted for NO_X_		1.09 (1.03–1.15)**	1.09 (1.01–1.17)*	1.06 (0.99–1.14)	1.10 (1.05–1.16)**

^a^ Adjusted for gender (adult and child), age (adult and child), education (adult), smoking (child) and passive smoking (household).

*P<0.05

** p<0.01

**Fig 2:**
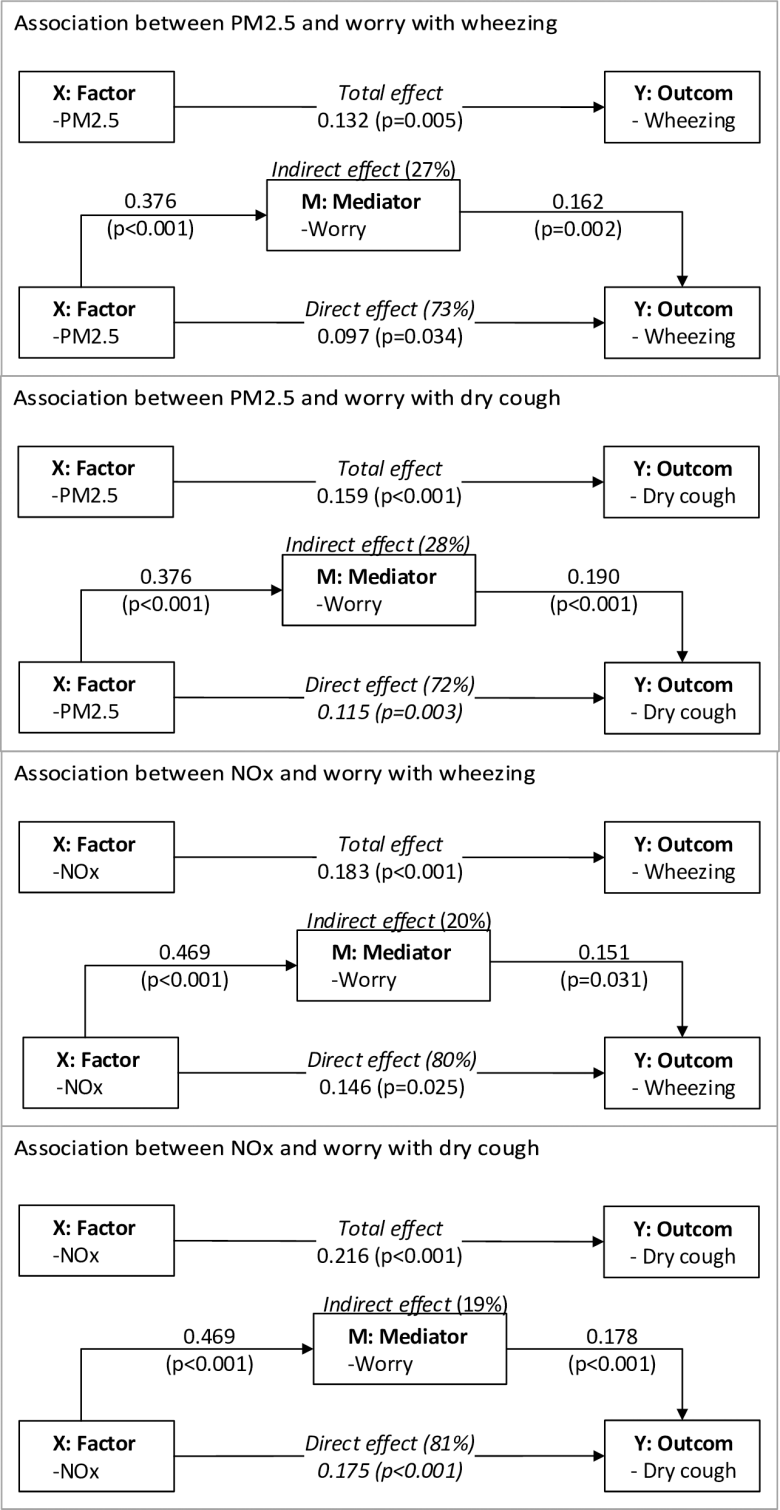
Mediation effects (worry) in exposure (X)–response (Y) models and mediation test after controlling for gender (adult and child), age (adult and child), education (adult), smoking (child) and passive smoking (household). The coefficients were made comparable across the equations.

Fig 2 illustrates the mediation model. The coefficients were made comparable across the equations. Parental worry significantly mediated the association of PM_2.5_ and NO_X_ on wheezing (27% and 20% respectively) and dry cough (28% and 19% respectively).

## Discussion

This study shows an association between PM_2.5_ exposure from the industry and self-reported high blood pressure in adults. We found an inversely association between NO_X_ exposure and cardiovascular diseases in adults. This is an unexpected finding. We found no explanation for this inversely association. In children a high PM_2.5_ and NO_X_ exposure was associated with an excess of wheezing and dry cough. Among children the association between exposure and health outcomes was mediated for 19%-28% by parental worry about local industry.

Comparable studies about self-reported health and industry-related air pollution are rare. In 2012 a cross-sectional questionnaire study among about 600 children between 7 and 13 years was conducted in the same area which showed similar associations between PM_2.5_ and NOx (per μg/m3) exposure, as predicted by an exposure dispersion model, and the presence of respiratory symptoms [26]. However no significant associations were found between the additional exposure to PM_2.5_ or NOx and respiratory symptoms except for PM_2.5_ and dry cough (OR 1.40, 95%CI 1.00 to 1.94). Due to the smaller study population in the study in 2012 compared to the current study (594 vs 1099) associations of similar magnitude lacked sufficient power. To the best of our knowledge there are no any other studies which describe the association of modelled PM_2.5_ or NO_X_ exposure from industry and self-reported symptoms. Other studies have compared populations living in industrial areas with control areas or have relied on emission information instead of modelled exposure patterns.

Epidemiological studies have supported the importance of risk and odour perception mechanisms for explaining the increased occurrence of self-reported health symptoms among residents living close to chemical industry [11–14]. However, to the best of our knowledge, there are no studies with a formal mediation analysis [23] to estimate the magnitude of the mediating effect of risk perception in associations between industry-related air pollution and self-reported health complaints. Our study showed that risk perception did not mediate associations between air pollution and presence of chronic diseases among adults. However, parental worry was indirectly accounting for 19%-28% of the magnitude of associations between air pollution and respiratory symptoms among their children. This is an important finding and it may be hypothesized that parents are less worried about the environmental impact on their own health than on the health of their children.

In our cross-sectional study it is not possible to disentangle the direction of the interrelations among risk perception, odour annoyance and health symptoms. Risk perception may contribute to odour annoyance and health symptoms. Odour annoyance can, in turn, be expected to affect risk perception and health symptoms. Health symptoms can also sensitize people to perceive odours and increase risk perception. In our study parents were asked if their child had respiratory symptoms. An alternative approach could have been asked for only the presence of a physician-based diagnosis of respiratory symptoms. It could be hypothesized that an objective diagnosis could attenuate the role of the mediator risk perception. However, over-diagnoses is also a potential problem as parents with a higher risk perception may have been more likely to visit their general practitioner.

In our study a strong correlation was found between the risk perception about local industry and odour pollution from local industry. Therefore, these two mediators cannot be modelled simultaneously because of collinearity. Risk perception about local industry was taken as mediator instead of odour annoyance by local industry because of its higher prevalence.

The direction of the mediating role of risk perception is important. If worry mediates the health outcomes and emissions from industries are reduced, then it is wisely also to communicate this in order to reduce worry and therefore decrease the prevalence of (self-reported) symptoms. Further research about different measurements of risk perception can give more insight into their mediating role in associations between environmental exposure and health complaints.

This study has certain strengths and limitations. First, there are different methods available for formally testing the presence of mediation. Our study used the most common method, namely the causal steps approach by Baron and Kenny [23]. Although commonly used, it must be recognized that the method has limitations. The method cannot deal well with interactions between the moderators (e.g. gender, age and education) and exposure or mediator. In our study there was no interaction of importance between the moderators and exposure or mediator. Thus, we feel that the use of this mediation method is justified.

Second, exposure to air pollution was based on a validated dispersion model. Good agreement was found for both SO_X_ and NO_X_ in the spatial patterns for the OPS dispersion model [27]. A linked problem is the use of two components as indicators for the exposure to air pollution. A variety of components were emitted by the industry in the industrial area. The exposure of each component may vary by locality. Because of the high correlation between the different components, it was not possible to single out association specific to a particular exposure component.

Third, the presence of the industry in the neighbourhood can be seen as a threat to residents’ health. Families with asthmatic children may avoid living near a chemical plant or move away. Therefore, migration bias may have decreased the presence of health symptoms in our study population and, therefore, may have attenuated the reported associations.

## Conclusions

In this cross-sectional study PM_2.5_ exposure from an area with heavy industry was related to reported high blood pressure in adults and NO_X_ exposure was inversely related to cardiovascular diseases in adults. In children a higher PM_2.5_ and NO_X_ concentration was associated with an excess of wheezing and dry cough. There was an important mediation role of worry about industry in the associations between parental worry and children’s respiratory symptoms. This study demonstrated that worry about industry cannot be ignored in studies about air pollution from industry and self-reported health.

## Supporting information

S1 Fig Modelled NO_X_ isoconcentration contours (μg/m^3^), mean annual exposure without background concentration. **Map reprinted from Kadaster in the Netherlands** [25] **under a CC-BY-4.0 license, 2017.**
(TIF)

## References

[1] Mannucci PM, Harari S, Martinelli I, Franchini M. Effects on health of air pollution: a narrative review. Intern Emerg Med. Springer Milan; 2015;10: 657–662. doi: 10.1007/s11739-015-1276-7 26134027 10.1007/s11739-015-1276-7

[2] Liang R, Zhang B, Zhao X, Ruan Y, Lian H, Fan Z. Effect of exposure to PM2.5 on blood pressure: a systematic review and meta-analysis. J Hypertens. 2014;32: 2130–41. doi: 10.1097/HJH.0000000000000342 25250520 10.1097/HJH.0000000000000342

[3] Kurt OK, Zhang J, Pinkerton KE. Pulmonary health effects of air pollution. Pulm Med. 2016;22: 138–143. doi: 10.1097/MCP.0000000000000248 26761628 10.1097/MCP.0000000000000248 PMC4776742

[4] Pascal L, Pascal M, Stempfelet M, Goria S, Declercq C. Ecological study on hospitalizations for cancer, cardiovascular, and respiratory diseases in the industrial area of Etang-de-Berre in the South of France. J Environ Public Health. 2013; 13 doi: 10.1155/2013/328737 10.1155/2013/328737 PMC3706020 23864868

[5] Smargiassi A, Goldberg MS, Wheeler AJ, Plante C, Valois MF, Mallach G, et al Associations between personal exposure to air pollutants and lung function tests and cardiovascular indices among children with asthma living near an industrial complex and petroleum refineries. Environ Res. 2014;132: 38–45. doi: 10.1016/j.envres.2014.03.030 24742726 10.1016/j.envres.2014.03.030

[6] Nirel R, Maimon N, Fireman E, Agami S, Eyal A, Peretz A. Respiratory hospitalizations of children living near a hazardous industrial site adjusted for prevalent dust: A case-control study. Int J Hyg Environ Health. 2015;218: 273–279. doi: 10.1016/j.ijheh.2014.12.003 25547415 10.1016/j.ijheh.2014.12.003

[7] Wichmann FA, Müller A, Busi LE, Cianni N, Massolo L, Schlink U, et al Increased asthma and respiratory symptoms in children exposed to petrochemical pollution. J Allergy Clin Immunol. 2009;123: 632–638. doi: 10.1016/j.jaci.2008.09.052 19111332 10.1016/j.jaci.2008.09.052

[8] Rovira E, Cuadras A, Aguilar X, Esteban L, Borràs-Santos A, Zock J-P, et al Asthma, respiratory symptoms and lung function in children living near a petrochemical site. Environ Res. 2014;133: 156–163. doi: 10.1016/j.envres.2014.05.022 24949814 10.1016/j.envres.2014.05.022

[9] White N, TeWaterNaude J, van der Walt A, Ravenscroft G, Roberts W, Ehrlich R. Meteorologically estimated exposure but not distance predicts asthma symptoms in schoolchildren in the environs of a petrochemical refinery: a cross-sectional study. Environ Health. 2009;8: 45 doi: 10.1186/1476-069X-8-45 19781087 10.1186/1476-069X-8-45 PMC2764638

[10] Moraes ACL De, Ignotti E, Netto PA, Jacobson LDSV, Castro H, Hacon SDS. Wheezing in children and adolescents living next to a petrochemical plant in Rio Grande do Norte, Brazil. J Pediatr (Rio J). 2010;86: 337–344. doi: 10.2223/JPED.2020 20711548 10.2223/JPED.2020

[11] Luginaah IN, Taylor SM, Elliott SJ, Eyles JD. Community reappraisal of the perceived health effects of a petroleum refinery. Soc Sci Med. 2002;55: 47–61. 12137188 10.1016/s0277-9536(01)00206-4

[12] Axelsson G, Stockfelt L, Andersson E, Gidlof-Gunnarsson A, Sallsten G, Barregard L. Annoyance and worry in a petrochemical industrial area—Prevalence, time trends and risk indicators. Int J Environ Res Public Health. 2013;10: 1418–1438. doi: 10.3390/ijerph10041418 23552810 10.3390/ijerph10041418 PMC3709326

[13] Atari DO, Luginaah IN, Fung K. The relationship between odour annoyance scores and modelled ambient air pollution in Sarnia, “Chemical Valley”, Ontario. Int J Environ Res Public Health. 2009;6: 2655–2675. doi: 10.3390/ijerph6102655 20054461 10.3390/ijerph6102655 PMC2790099

[14] Stenlund T, Lidén E, Andersson K, Garvill J, Nordin S. Annoyance and health symptoms and their influencing factors: A population-based air pollution intervention study. Public Health. 2009;123: 339–345. doi: 10.1016/j.puhe.2008.12.021 19344922 10.1016/j.puhe.2008.12.021

[15] Boffetta P, Nyberg F. Contribution of environmental factors to cancer risk. British Medical Bulletin. 2003 pp. 71–94. doi: 10.1093/bmp/ldg023 10.1093/bmp/ldg023 14757710

[16] Pollutant Release and Transfer Register [Internet]. [cited 19 Oct 2017]. Available: http://www.emissieregistratie.nl/

[17] Fayers PM, Sprangers MA. Understanding self-rated health. Lancet. 2002;359: 187–188. doi: 10.1016/S0140-6736(02)07466-4 11812551 10.1016/S0140-6736(02)07466-4

[18] Botterweck A, Frenken F, Janssen S, Rozendaal L, Vree M De, Otten F. Plausibiliteit nieuwe metingen algemene gezondheid en leefstijlen 2001 Statistics Netherlands (CBS), Heerlen, The Netherlands; 2003.

[19] Van Overveld AJP, Franssen EAM. Naar een monitor voor beleving van de leefomgeving: Handreiking en vragenlijst voor GGD’en Report 609300010/2009. The National Institute for Public Health and the Environment (RIMV), Bilthoven, The Netherlands; 2009.

[20] Asher MI, Keil U, Anderson HR, Beasley R, Crane J, Martinez F, et al International study of asthma and allergies in childhood (ISAAC): Rationale and methods. Eur Respir J. 1995;8: 483–491. doi: 10.1183/09031936.95.08030483 7789502 10.1183/09031936.95.08030483

[21] Beasley R. Worldwide variation in prevalence of symptoms of asthma, allergic rhinoconjunctivitis, and atopic eczema: ISAAC. Lancet. 1998;351: 1225–1232. doi: 10.1016/S0140-6736(97)07302-9 9643741

[22] Lai CKW, Beasley R, Crane J, Foliaki S, Shah J, Weiland S. Global variation in the prevalence and severity of asthma symptoms: phase three of the International Study of Asthma and Allergies in Childhood (ISAAC). Thorax. 2009;64: 476–483. doi: 10.1136/thx.2008.106609 19237391 10.1136/thx.2008.106609

[23] Baron RM, Kenny DA. The Moderator-Mediator Variable Distinction in Social The Moderator-Mediator Variable Distinction in Social Psychological Research: Conceptual, Strategic, and Statistical Considerations. J Pers Soc Psychol. 1986;51: 1173–1182. doi: 10.1037/0022-3514.51.6.1173 3806354 10.1037//0022-3514.51.6.1173

[24] MacKinnon DP, Dwyer JH. Estimating mediated effects in prevention studies. Eval Rev. 1993;17: 144–158.

[25] Kadaster, The Nederlands [Internet]. Available: https://www.pdok.nl/nl/over-pdok/uw-speciale-aandacht/copyright

[26] Bergstra AD, Brunekreef B, Burdorf A. The effect of industry-related air pollution on lung function and respiratory symptoms in school children. Environ Heal. 2018;17: 1–9. doi: 10.1186/s12940-018-0373-2 29587756 10.1186/s12940-018-0373-2 PMC5872550

[27] Van Jaarsveld JA. The operational priority substances model: Description and validation of OPS-Pro 4.1 Report 500045001/2004. The National Institute for Public Health and the Environment (RIMV), Bilthoven, The Netherlands; 2004.

