# Supplementary figures and images for: The mediating role of risk perception in the association between industry-related air pollution and health
Source: PLoS One. 2018 May 3;13(5):e0196783. doi: 10.1371/journal.pone.0196783 (PMC5933722; doi:10.1371/journal.pone.0196783)

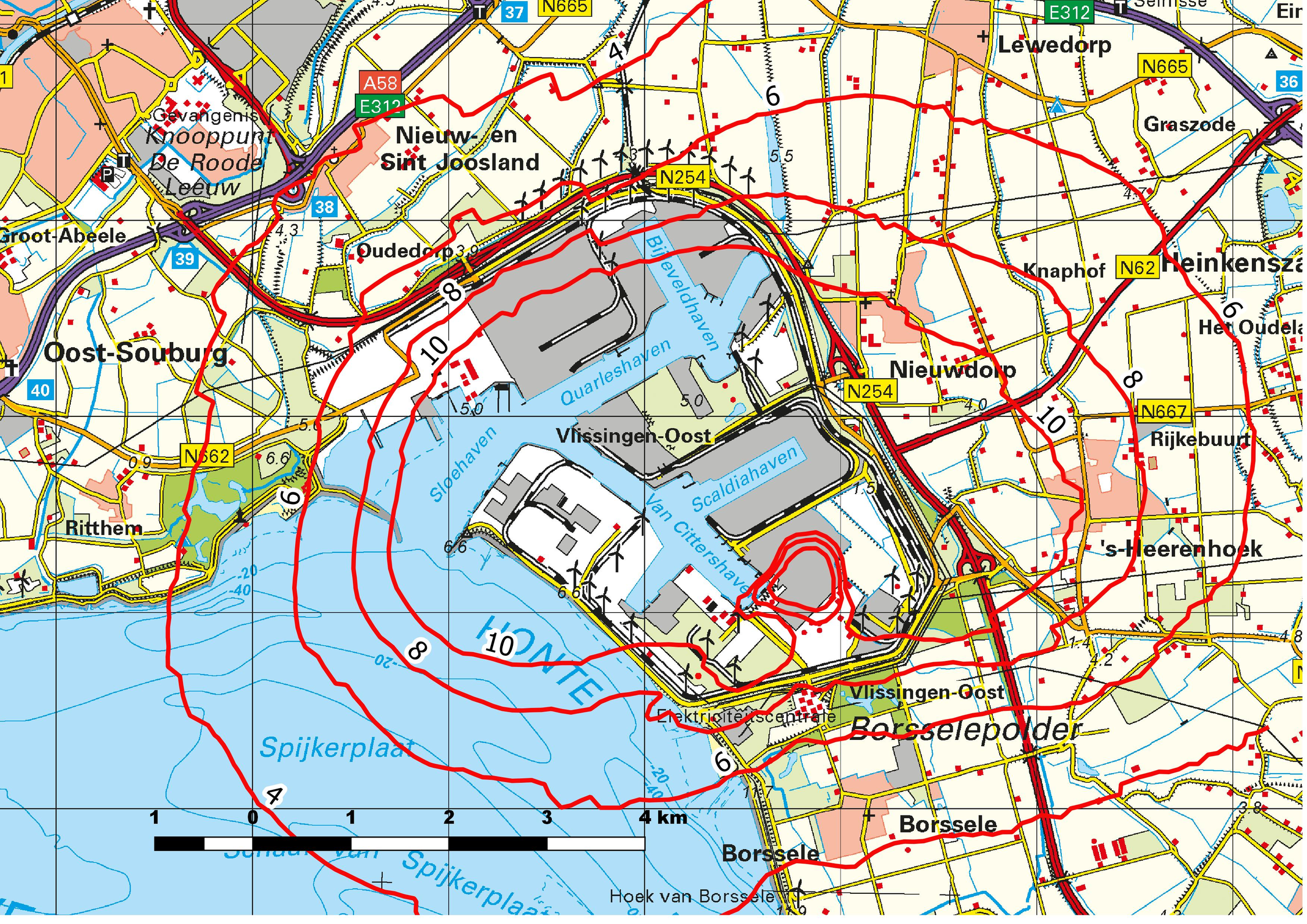

Supplement: S1 Fig — Map reprinted from Kadaster in the Netherlands [25] under a CC-BY-4.0 license, 2017. (TIF) [file pone.0196783.s001.tif]
